# How to Evaluate Health-Related Quality of Life and Its Association with Medication Adherence in Pulmonary Tuberculosis – Designing a Prospective Observational Study in South Africa

**DOI:** 10.3389/fphar.2016.00125

**Published:** 2016-05-31

**Authors:** Tanja Kastien-Hilka, Bernd Rosenkranz, Bryan Bennett, Edina Sinanovic, Matthias Schwenkglenks

**Affiliations:** ^1^Swiss Tropical and Public Health InstituteBasel, Switzerland; ^2^University of BaselBasel, Switzerland; ^3^Health Economics Unit, Faculty of Health Sciences, University of Cape TownCape Town, South Africa; ^4^Division of Clinical Pharmacology, Faculty of Medicine and Health Sciences, Stellenbosch UniversityCape Town, South Africa; ^5^Fundisa African Academy of Medicines DevelopmentCape Town, South Africa; ^6^Patient Centered Outcomes, Adelphi ValuesBollington, UK; ^7^Institute of Pharmaceutical Medicine, University of BaselBasel, Switzerland; ^8^Epidemiology, Biostatistics and Prevention Institute, University of ZürichZürich, Switzerland

**Keywords:** health-related quality of life, adherence, tuberculosis, study design, South Africa

## Abstract

**Introduction:** Health-related quality of life (HRQOL) has become an important measure to identify and shape effective and patient-relevant healthcare interventions innovations through outcomes. Adherence to tuberculosis (TB) treatment is a public health concern. The main objective of this research is to develop a study design for evaluation of HRQOL and its association with medication adherence in TB in South Africa.

**Methodology:** A conceptual framework for HRQOL in TB has been developed to identify Patient-Reported Outcomes and Quality of Life Database (PROQOLID), (n.d.) measures for HRQOL and adherence and to generate an endpoint model. Two generic (SF-12 and EQ-5D-5L), one disease-specific (St. George’s Respiratory Questionnaire) and one condition-specific (Hospital Anxiety and Depression Scale) measure for HRQOL and Morisky Medication Adherence Scale for adherence assessment were identified. All measures are applied in a longitudinal multi-center study at five data collection time points during standard TB treatment. Statistical analysis includes multivariable analysis. Change over time in the physical component score of SF-12 is defined as primary endpoint. Sample size estimation based thereupon has led to a recruitment target of 96 patients. This study is on-going.

**Discussion:** This is the first longitudinal study in South Africa which evaluates HRQOL and its association with medication adherence in TB in a comprehensive manner. Results will help to improve current treatment programs and medication adherence and will support the identification of sustainable health innovations in TB, determining the value of new products, and supporting decision making with regard to health policy and pricing.

## Introduction

According to the World Health Organization (WHO), health has a multi-dimensional nature and comprises physical, mental, and social health domains. (World Health Organization, 1948) Health-related quality of life (HRQOL) is a patient-reported outcome (PRO) parameter which refers to the multi-dimensional nature of health directly from the patient perspective. Tuberculosis (TB) places a significant burden on the health system of South Africa, which has the highest prevalence and incidence rates of all 22 high-burden TB countries worldwide (World Health Organization [WHO], 2015). Treatment is available in South Africa, however, the incidence rate is only slowly decreasing and the relapse rate is high. Both epidemiological parameters may be influenced by HRQOL in TB. They may also be additionally affected through inadequate treatment adherence although direct observed treatment (DOT) has been introduced for supervised treatment monitoring. To study these associations, further research is required. The study presented here intends to evaluate HRQOL and its association with medication adherence in TB in South Africa. To understand the impact of HRQOL in TB and determinants of medication adherence, we first conducted a systematic review of the available literature (Kastien-Hilka et al., 2016). In brief, active TB impacts HRQOL significantly across different health settings; the impairment affects physical, emotional, psychological, and social as well as economical aspects. Although, standard TB treatment improves all health domains, psychological well-being and social functioning remained impaired in microbiologically cured patients after treatment (Muniyandi et al., 2007; Pasipanodya et al., 2007; Weis and Pasipanodya, 2010; Ralph et al., 2013; PROQOLID). Most published studies on HRQOL in TB had a cross-sectional design (Atif et al., 2014b), however, to fully understand the impairment of HRQOL, TB impact needs to be assessed over the complete treatment period (Brown et al., 2015). In our systematic review we identified only 10 studies which have followed a longitudinal approach; none of them in a South African environment (Chamla, 2004; Marra et al., 2008; Dhuria et al., 2009; Maguire et al., 2009; Kruijshaar et al., 2010; Balgude and Sontakke, 2012; Aggarwal et al., 2013; Ralph et al., 2013; Atif et al., 2014a; Mamani et al., 2014). Similar factors as for HRQOL impact medication adherence (World Health Organization [WHO], 2003; Kastien-Hilka et al., 2016). Medication adherence “the process by which patients take their medications as prescribed” (Vrijens et al., 2012). It comprises the initiation of the treatment (first dose), implementation of the prescribed dosing regime, and discontinuation of the therapy (end of therapy), and the persistence (time from initiation until discontinuation; Vrijens et al., 2012; Lam and Fresco, 2015) Medication adherence is crucial to reach clinical targets and the causes of non-adherence are multi-factorial (Fairman and Motheral, 2000; Lam and Fresco, 2015). WHO stated that “increasing the effectiveness of adherence interventions may have a far greater impact on the health of the population than any improvement in specific medical treatment” (World Health Organization [WHO], 2003). Both HRQOL and adherence are related to the patient (Cote et al., 2003). A change in adherence is followed by a change in HRQOL and a relationship between adherence and HRQOL is existing (Cote et al., 2003; Kastien-Hilka et al., 2016).

We perform a longitudinal study for evaluating patient-reported HRQOL and its association with medication adherence in TB in South Africa. This study is specific to TB and transferability to other diseases are beyond the scope of this research. The study will provide information on the health status of TB patients and their HRQOL; HRQOL outcomes will allow understanding which health domains are most impacted by TB. Adherence behavior will be observed from a patient perspective. This will allow us to identify which health aspects of TB are influencing adherence and which health domains are impacted by non-adherence. Since the study is taking place among patients from South Africa, the cultural and socio-demographic impact on TB are respected. This article describes the study design and rationale.

## Materials and Stepwise Procedures

### Study Aim and Outcomes

The main aim of the study is to evaluate HRQOL and medication adherence in active TB patients in South Africa. Specific study outcomes comprise following: to assess TB impact on HRQOL and its longitudinal changes during standard TB treatment; to understand patient-reported medication adherence and its longitudinal changes during standard TB treatment; to evaluate any influence of socio-demographic aspects on HRQOL and adherence with regard to gender, age, education, work status and co-morbidities in sub-group analysis; to assess any association between changes in HRQOL and changes in adherence and sputum smear results; to understand if an association between HRQOL and adherence during standard TB treatment is existing and which health domains are involved.

The following preparatory steps were undertaken during the design phase of the study and are described in detail below: conceptualization of HRQOL in TB; identification of patient-reported measures applied in HRQOL evaluation and in studies of adherence to anti-TB standard treatment; selection of HRQOL and adherence measures capturing all relevant health aspects of TB; definition of data collection points for HRQOL and adherence; identification of a primary endpoint on basis of HRQOL and development of an endpoint model; selection of study setting; definition of study participant inclusion and exclusion criteria; development of a rationale for sample size determination; definition of statistical analysis (PROQOLID; EuroQol).

### Conceptualization of HRQOL in TB

**FIGURE 1:**
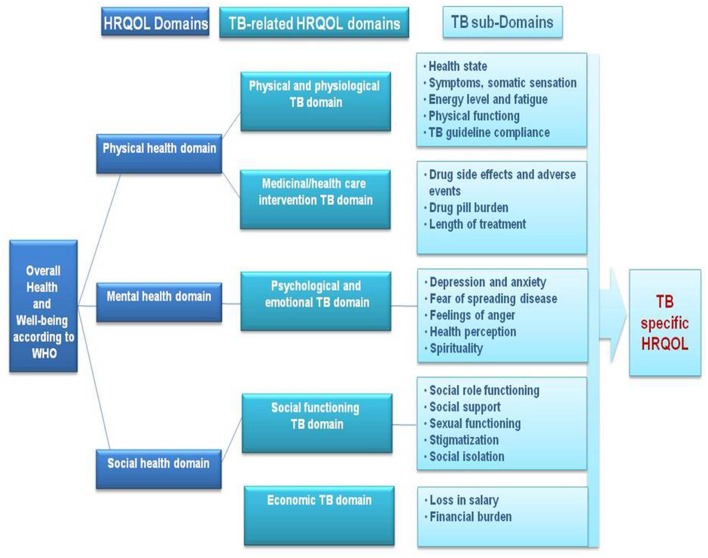
**Conceptual framework of HRQOL in TB, developed based on systematic review results**.

The results of the literature review identified that TB impacts physical, emotional, psychological, social and economic domains of HRQOL, with residual impairment still present in each of these domains even after treatment. Based on these results, a conceptual framework of HRQOL in TB has been developed (**Figure 1**).

The physical health domain is mainly described by TB-related physical and physiological aspects which comprise disease symptoms, health status, fatigue and physical functioning. There are also influences of TB treatment due to side effects, pill burden, and duration of treatment. The mental health domain is mainly impacted by the psychological and emotional aspects related to TB which include depression and anxiety, fear of spreading TB, feelings of anger, health perception of the patient, and patient’s spirituality. The social health domain we defined by the social functioning of the patient and the economic burden related to TB. Social functioning is mainly impacted by social role, social support, sexual functioning, stigmatization, and social isolation. The economic burden is influenced by a loss in salary and any financial burden related to being ill.

### Identification of HRQOL and Adherence PRO Measures in TB

The systematic review yielded 38 studies which applied PRO measures in HRQOL and adherence: two systematic reviews, 21 cross-sectional studies, 14 longitudinal studies, and one qualitative study (Table A1 in appendix).

Based on the identified studies, 43 HRQOL and three adherence PRO measures were extracted, including 34 reported measures from the systematic reviews of Bauer et al. (2013) and 17 different HRQOL instruments from Guo et al. (2009; Tables A2 and A3 in appendix). Overall, the most frequently applied HRQOL instrument in TB was the generic Short-Form 36 (SF-36), followed by World Health Organization Quality of Life – BREF (WHOQOL-BREF), EuroQol-5D (EQ-5D) and disease-specific instruments, namely the Beck Depression Inventory (BDI), Kessler-10 (K-10) and St. George’s Respiratory Questionnaire (SGRQ). Our systematic review identified 10 studies applying a longitudinal design in HRQOL evaluation in TB (Chamla, 2004; Marra et al., 2008; Dhuria et al., 2009; Maguire et al., 2009; Kruijshaar et al., 2010; Balgude and Sontakke, 2012; Aggarwal et al., 2013; Ralph et al., 2013; Atif et al., 2014a; Mamani et al., 2014). Consistent with the results of the current review, the SF-36 was applied more often than other PROs to observe longitudinal changes. Next in frequency were the WHOQOL-BREF, SGRQ, EQ-5D, BDI, as well as the State-Trait Anxiety Short Form (STAI-6) and Center for Epidemiologic Studies Depression Scale (CES-D). In addition to our literature search, we accessed the database PROQOLID for HRQOL instruments which are applied in pulmonary and psychiatric illnesses and linguistically validated for application in English language in South Africa (PROQOLID). This identified 17 measures (Table A4 in appendix).

### Rationale for Selection of HRQOL and Adherence PRO Measures

A systematic use of generic HRQOL measures in all diseases supports consistent value judgments and transparent reimbursement decision making (European Network for Health Technology Assessment (eunethta), 2013). Two types of PRO measures are applied in HRQOL evaluations: generic measures and disease- or condition-specific measures. Generic measures have the advantage of allowing comparison of quality of life outcomes across different diseases and can accommodate effects of co-morbid conditions; they are often applied to inform health policy maker about allocation of resources (European Network for Health Technology Assessment (eunethta), 2013). In contrast to generic measures, disease-specific instruments comprise disease specific health aspects (Doward et al., 2010; Deshpande et al., 2011; European Network for Health Technology Assessment (eunethta), 2013). Disease- and condition-specific measures may be more sensitive to changes in HRQOL and may better detect important disease-related changes over time. Disease- and condition-specific HRQOL information may be complementary to generic HRQOL measures, specifically when no effect is observed with generic measures. However, a recognized and validated TB-specific HRQOL instrument currently does not exist. We therefore followed the approach to select generic and disease- and condition-specific measures which capture all major HRQOL-related aspects of TB based on our conceptual framework.

All 43 measures applied in HRQOL studies and three adherence instruments identified by our literature search as well as all 17 instruments from the PROQOLID database search were reviewed. Measures were selected in order to capture physical, mental, and social health issues of TB. In addition to consistency with our conceptual framework, selection required that the respective measures had been validated in TB and linguistically validated for English in South Africa. Based on these criteria, we finally selected the SGRQ as a disease-specific measure and the Hospital Anxiety and Depression Scale (HADS) as a condition-specific measure. The SGRQ is recommended as a preferred instrument by the European Medicines Agency [EMA] (2012) for evaluating quality of life in COPD patients. As a measure specifically for respiratory diseases, it captures important health aspects which can be applied to TB. HADS was selected as it allows evaluation of anxiety and of depression. As a preference-based and a profile measure, we selected the EQ-5D-5L and SF-12. Notably, the SF-12 has previously been used in pulmonary TB patients in South Africa for HRQOL assessment (Louw et al., 2012).

An ideal adherence measure to assess overall adherence to medication including initiation, persistance, implementation, and discontinuation does not exist; therefore, a multi-measure approach may be considered where direct and indirect adherence measure are combined (Fairman and Motheral, 2000). Direct measures such as drug determination in biological fluids or direct patient observation are often regarded as being the most reliable and accurate. However, individual changes in drug metabolism and multidrug intake affect monitoring of plasma levels, and patient observation is limited to inpatient settings and clinical trials (Fairman and Motheral, 2000; Nguyen et al., 2014; Lam and Fresco, 2015). Indirect measures comprise medication monitoring (such as pill counting) and self-reported measures; however, medication monitoring is cost intensive and self-reported measures may be less reliable due to patient recall, underreported non-adherence and memory bias issues (Fairman and Motheral, 2000; Lam and Fresco, 2015; Stirratt et al., 2015). Vrijens et al. (2012) defined adherence measures for the quantitative assessment of initiation, persistance, and implementation. Both, initiation and persistance are time-to-event variables and should be assessed by Kaplan–Meier curves, median persistence or proportion of persistent patients at a given time point. Implementation is assessed by a summary statistic such as the proportion of prescribed drug taken or longitudinally by electronic databases of drug dosing histories. Each adherence measure has its advantages and disadvantages and the selection of adherence measures depends on the attributes, research objectives and available resources of each study (Lam and Fresco, 2015). The present study focuses on the measurement of HRQOL and adherence in a real life middle-income setting in a township of South Africa. The study is purely observational, with no influence on treatment and follows patients during their TB care. The available resources of the study and the study environment do not allow application of direct adherence measures although they would in principle be the best instruments to measure patient’s medication taking behavior (Nguyen et al., 2014); Since DOT is established in South Africa, we decided to apply a self-reported measure as it allows to identify specific reasons for being non-adherent (Nguyen et al., 2014; Lam and Fresco, 2015). Further, a self-reported measure is best applicable to the study environment and study settings as it is cost-effective, with a minimum burden to the patient, easy to administer and flexible in timing and mode of administration. Self-reported adherence measures have additional advantages; they inform about non-adherence before adverse clinical outcomes develop and about adherence determinants such as psychosocial factors (Stirratt et al., 2015). A gold standard for self-reported, questionnaire-based adherence measurement does not exist (Fairman and Motheral, 2000; Nguyen et al., 2014). The Morisky Medication Adherence Scale (MMAS) is widely used, reliable and valid and has been applied in TB before (McInerney et al., 2007; Corless et al., 2009; Lam and Fresco, 2015; Stirratt et al., 2015). MMAS allows the evaluation of the concept of medication-taking behaviors, barriers to adherence, and beliefs associated with adherence (Lam and Fresco, 2015) including forgetfulness and adverse events (Nguyen et al., 2014). Based on the taxonomy of adherence by Vrijens et al. (2012) MMAS covers the implementation and discontinuation phase of adherence. The assessment of self-reported scales includes the correlation with an objective adherence measure, and MMAS was correlated with pharmacy records and clinical outcomes (blood pressure control) as comparison measure of adherence (Nguyen et al., 2014).

All four HRQOL measures and the adherence measures are reliable and validated (PROQOLID; Morisky et al., 2008; Lam and Fresco, 2015), and the HRQOL measures were linguistically validated for English in South Africa (Euroqol; PROQOLID; St. George’s University of London, 2012). All selected measures are described in detail in the appendix.

### Rationale for Study Endpoint Model

An endpoint model for the present study was based on measurement concepts as reported by Burke from the US FDA (Burke, 2012). Treatment benefit measurements comprise four different concepts: disease-defining concepts, proximal disease and distal disease impact concepts, and disease impact on general life concepts.

**FIGURE 2:**
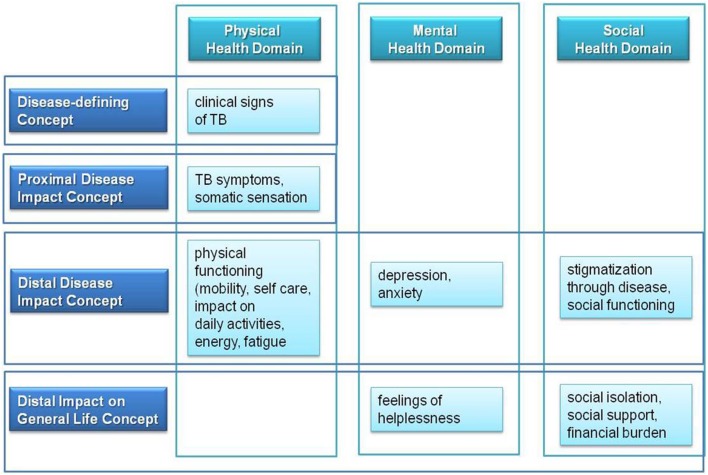
**Endpoint concept for TB based on major HRQOL health domains and on benefit treatment measurement concepts**.

**FIGURE 3:**
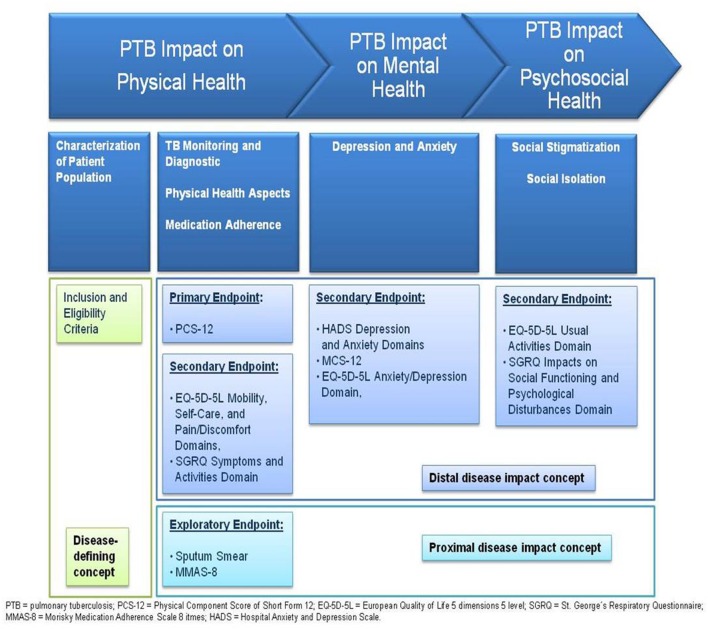
**Final endpoint model for evaluation of HRQOL and treatment adherence in PTB, respecting HRQOL domains and disease-defining as well as proximal and distalmeasurement concepts**.

Disease-defining concepts describe clinical signs of a disease. The impact of these disease-specific clinical signs is reflected in proximal disease impact concepts of disease symptoms and somatic sensation. Both disease-defining and proximal disease impact concepts reflect some aspects of the physical health domain of the conceptual psychometric framework of HRQOL. Mental, social, and psychosocial domains of HRQOL are represented through the distal disease impact concepts. Distal disease impact concepts include physical, psychological and social functioning, thereby reflecting additional aspects of the physical health domain. The disease impact on general life concept comprises aspects which result from impairment in distal disease impact concepts, such as impairment of social role functioning, economic burden and quality of life in general. Based on the benefit treatment measurement concept of Burke (Burke, 2012) and based on the psychometric concept of TB-specific HRQOL, we developed following endpoint concept for TB (**Figure 2**):

The selected HRQOL and adherence measures were integrated into the endpoint concept. The physical health domain of HRQOL is evaluated by the physical component score (PCS) of the SF-12 (PCS-12). The change between end of treatment (EOT) and baseline in the PCS-12 score is defined as the primary study endpoint. The EQ-5D-5L physical domain and SGRQ symptoms domain scores represent secondary endpoints. The mental health domain of HRQOL is assessed by the mental component score of the SF-12 (MCS-12), the EQ-5D-5L mental domain, SGRQ activities domain, and HADS. The (psycho-) social health domain of HRQOL is evaluated by SGRQ impacts on social activities domain. Other endpoints comprise clinical data (sputum smear) for presence of active TB and treatment monitoring as well as medication adherence by application of MMAS-8. **Table 1** provides an overview of endpoints selected and related instruments applied. The final endpoint model for evaluation of HRQOL and adherence in TB is presented in **Figure 3.**

**Table 1:** Lists primary, secondary, and other endpoints included in the study in order to evaluate longitudinal changes in HRQOL and adherence.

Endpoint (mean difference EOT minus BL)	PRO measure
Primary endpoint	SF-12 PCS-12
Secondary endpoint	SF-12 MCS-12
	EQ-5D-5L
	SGRQ
	HADS
Other endpoint	Clinical data (sputum smear)
	MMAS-8


### Study Design and Study Setting

The study is observational in nature with a longitudinal design including repeated measures of HRQOL and adherence per study participant. Multiple health facilities are included and the study has thereby a multi-center design. The highest TB burden in Cape Town is recorded in the Khayelitsha sub-district. Six health facilities with the highest TB caseloads per month were selected. Each health facility has an outpatient TB department. The caseloads of TB cases for each selected health facility were provided through the Western Cape Province.

### Patient Population and Participant Recruitment

The patient population comprises patients diagnosed with active pulmonary TB. TB patients are eligible if their age is 18 years or older and if they have not received standard TB treatment prior to this study. Patients are excluded if they are diagnosed with multidrug-resistant TB (MDR-TB) and extensively drug-resistant TB (XDR-TB), and/or with HIV co-infection. The eligibility status of each patient is subject to verification by the responsible TB nurse of each health facility. Based on a patient information document, eligible patients are informed about nature, purpose, potential risks, and benefits of the study. Patients have the opportunity to decline their participation or to withdraw from the study at any time point. If an eligible patient agrees to participate in the study, a written informed consent is obtained before study start. The sample population is reached by pooling eligible patients from all study sites.

### Study Procedure

Eligible participants receive a 6 months standard TB treatment with rifampicin, isoniazid, ethambutol, and pyrazinamide at each health clinic or health center. The drug treatment is local standard care according to the national TB guideline and DOT is applied (Department of Health Republic of South Africa, 2014). The treatment is not influenced by this non-interventional research study. The study evaluates HRQOL and adherence based on questionnaires completed by patients, who are treated and managed in the health facility independently from this study. Most studies evaluating HRQOL longitudinally included data collection before treatment or at treatment start, at the switch from the intensive to continuous treatment phase and at EOT (Chamla, 2004; Marra et al., 2008; Dhuria et al., 2009; Maguire et al., 2009; Kruijshaar et al., 2010; Balgude and Sontakke, 2012; Aggarwal et al., 2013; Ralph et al., 2013; Atif et al., 2014a; Mamani et al., 2014). We decided on a tighter data collection regimen to monitor changes in HRQOL more closely and included five different time points over the 6 months treatment period: beginning of treatment (baseline) and follow-up visits 1–4 after 1, 2, 4, and 6 months (EOT).

Data collection starts when TB treatment is initiated; the four HRQOL measures and a socio-demographic questionnaire are applied at baseline (treatment start). The four HRQOL measures are repeatedly applied at all follow up visits 1–4 together with the MMAS. Data are collected based on combined completion of paper questionnaires and face-to-face interviews through trained field workers. Training of field workers is based on a Standard Operating Procedure (SOP) specifically written for this study. Questionnaires are scanned by the field worker for any missing responses. The patient is asked to provide an answer for the missing response unless the question was intentionally left unanswered. TB-related clinical data (sputum smear results) are collected from TB nurses when available during the treatment period for each patient.

### Data Management

All paper-based documents including the questionnaires and informed consent forms are stored at the University of Cape Town for a period of 10 years. The HRQOL, adherence and socio-demographic measures, and sputum smear results data are transferred to an electronic database independently by two research team members. Every third questionnaire is entered into a second database. Both databases are reconciled to minimize errors related to data entry. All questionnaire responses are scored according to each questionnaire’s manual instructions. Scored data are prepared for statistical analysis applying SPSS software.

### Rationale for Sample Size Determination

The primary endpoint is the PCS of SF-12. Walters (2004) compared four different methods for sample size estimation using SF-36 as primary endpoint. Since SF-12 has shown comparable results with SF-36, both measures are seen as similar and comparable and the methods discussed applicable to SF-12. We decided to select a method for sample size determination which is based on the mean difference. The estimation of mean difference in PCS-12 scores is defined as average mean difference score between EOT and baseline (BL). We therefore performed a literature search for studies applying SF-12 or SF-36 as an outcome measure during TB treatment, and found 10 studies presenting SF-12 and SF-36 scores (Dion et al., 2004; Guo et al., 2008; Marra et al., 2008; Babikako et al., 2010; Kruijshaar et al., 2010; Louw et al., 2012; Bauer et al., 2013; Atif et al., 2014a). Only two studies reported PCS-36 scores over the 6 months long TB treatment, and observed a change in mean score over time (baseline to EOT) with a mean difference of 4.0 (Babikako et al., 2010) and of 4.1 (Atif et al., 2014a). The minimal important difference (MID) for SF-36 and SF-12 is reported with >3 points by the questionnaire manual (Maruish and Kosinski, 2009). We therefore assumed a mean difference of 4.0 for our study. A standard deviation (SD) for PCS-12 was defined with the value 7 at EOT for PCS-36 (Atif et al., 2014a). Final sample size was calculated based on a mean difference of 4.0 and a SD of 7 for mean PCS-12, resulting in a standardized effect size of 0.57. Using the standardized effect size with a two-sided 5% level of significance and a 95% power yielded in an estimated sample size *n* = 80 subjects. We estimated an attrition rate of 20% leading to a final sample size *n* = 96 patients.

### Anticipated Results

Based on published HRQOL studies in TB, we expect an improvement in HRQOL during the course of treatment; especially physical health aspects are expected to improve. However, some residual physical impairment has previously been reported after treatment end, although patients’ were microbiologically cured. Reported mental and psychosocial health aspects included depression and anxiety, and social isolation and stigmatization. We expect to find these aspects of health impairment reflected in HRQOL outcomes at the beginning of treatment. As TB treatment requires a long-term dosing regimen, we expect a decreasing adherence behavior over the treatment course, although DOT is established and a requirement in South Africa. Potential pitfalls of our study are mainly driven by the infrastructure of the health setting in the township Khayelitsha, which, however, reflects South African reality. Patients have no regular access to mobile phones and interview dates for HRQOL and adherence interviews are difficult to organize; follow up with the patients is also difficult for the health facilities. We expect a number of missing interviews and interviews taken not at the required time points, albeit around the target interview dates. Our HRQOL and adherence measures are self-reported measures and reflect a subjective patient view. Related bias including over- and under-reporting of both concepts can be expected. This will be consistent with the individual patient’s TB condition. Experienced pitfalls for future studies will be discussed as lessons learned from our study.

### Statistical Analysis

All statistical analyses are conducted using IBM SPSS Version 22^®^. Descriptive statistics are calculated for socio-demographic descriptors of the patient population and all study outcomes. Study outcomes comprise the means of all HRQOL domain and total scores, adherence scores and sputum smear results, and related changes over time. Categorical variables are described by frequencies, proportions (%) and number of missing values. Continuous variables (HRQOL, adherence) are described by the mean and median as measures of central tendency, first and third quartiles, standard deviation (SD), minimum (Min) and maximum value (Max), and number of missing values. Continuous variables are checked for non-normality with q–q plots and histograms. Outliners are observed via boxplots; if applicable 5% trimmed means may be calculated. Ordinal variables representing responses to questionnaire items are described in the same way as categorical and/or continuous variables, depending on their properties. For example, responses to an item with three answer levels would be described in the same way as a categorical variable whereas responses to an 11 step (0–10) rating scale would be described in the same way as a continuous variable.

Change of each HRQOL measure and of the adherence measure between baseline and each follow up visit is calculated based on mean scores. Changes in mean scores between baseline and EOT are presented as boxplots. Differences between baseline and each follow up are examined based on significance testing; choice of tests considers the distribution of the variables involved. For distributions with no or only limited deviations from normality, the paired *t*-test is used, otherwise the Mann–Whitney *U*-test. The level of statistical significance is set at 5%, two-sided for both the paired *t*-test and Mann–Whitney *U*-test. Change in HRQOL mean scores over time (BL, visit 1–4) is further examined by two-way repeated measure analysis of variance (ANOVA), for all HRQOL instruments.

Responsiveness of HRQOL measures refers to their ability to detect important change over time in the concept measured, even if this change is small. Each change in mean score for each measure is compared with its reported MID. When the change in mean score is equal or above the MID, the change is interpreted as meaningful. Further, the effect size is calculated by dividing the change in mean score through the standard deviation at baseline (0.2, 0.5, and 0.8 represent small, medium, and large effect size; Cohen, 1988; Aggarwal et al., 2013). It is further observed which health domains improve, worsen, or remain unchanged over time based on change in mean scores. Hypothesis testing is applied to HRQOL, adherence and sputum smear outcomes. The hypotheses testing comprises following assumptions: HRQOL improves, adherence declines, and sputum smear results improve over time from baseline to EOT. Hypothesis testing is performed by repeated measures ANOVA if data are normally distributed or with the Kruskal–Wallis test for non-normally distributed data. Sub-group analyses are performed additionally, by gender, age, ethnicity, marital status, educational and work status, and co-morbidities.

Additional relationships are assessed for HRQOL, adherence, and sputum smear results, including sub-groups analyses according to socio-demographic data. First, the correlations between all health domains of all HRQOL measures at baseline are assessed by applying Pearson’s correlation coefficient or Spearman’s rho coefficient. A correlation matrix is presented. Second, it is examined how changes in HRQOL and changes in adherence are associated with changes in sputum smear results over time. Correlation coefficients are calculated and linear mixed regression models are estimated. Third, it is examined how changes in both HRQOL and adherence are linked with the socio-demographic variables by applying linear mixed regression models. A final analysis assesses how HRQOL and adherence are associated with each other, from baseline to EOT, by applying linear mixed regression models. Adjustments for multiple testing will be made where sensible. Related issues will be discussed.

## Discussion

The impact of TB on patients’ HRQOL has been reported in international studies including some conducted in high-burden TB countries but its longitudinal changes have not been assessed in South African TB patients. South Africa is the country with the highest prevalence and incidence rate in TB among all 22 high-burden countries (World Health Organization [WHO], 2015). Limited knowledge about adherence behavior during TB treatment is available, most from qualitative studies. Both outcomes, HRQOL and adherence, are assumed to impact each other but this association has not been studied longitudinally so far. The objective of this research is to perform a study evaluating HRQOL and its association with medication adherence in patients with active pulmonary tuberculosis in South Africa. We developed a conceptual framework for HRQOL in TB capturing all TB related physical, mental and psycho-social health aspects; based on a systematic literature search we found a total of 43 different HRQOL and three adherence measures which were applied in TB. Based on these findings we selected HRQOL and adherence measures which capture all HRQOL constructs in TB according to our conceptual framework. The rationale of PRO measure selection respected the use of generic HRQOL measures which allow comparison between different diseases and thus support consistent value judgment and transparency, e.g., in reimbursement decision making (European Network for Health Technology Assessment (eunethta), 2013). Further, we included disease- and condition-specific instruments which are more sensitive to change than generic instruments. This ultimately led to the selection of two generic measures (EQ-5D-5L and SF-12), one disease-specific (St. George Respiratory Questionnaire) and one condition-specific measure (HADS). As a patient-reported adherence instrument, we selected the MMAS. Validity and reliability of these selected PRO measures, including their linguistic validation for English for South Africa, is given. MID was identified from literature for each HRQOL measure to understand meaningful changes over time. Based on the conceptual framework and selection of PRO measures, an endpoint model with the change between EOT and baseline in the PCS of SF-12 defined as primary endpoint was developed. PCS score of SF-12 is selected as primary endpoint since TB has a major impact on the physical health. Based on the primary endpoint, the required sample size was determined to be 96 participants. The described protocol is specific to TB, however, the rationale for selection of HRQOL and adherence measures may be transferred to other studies applying PRO measures and might be useful for the development of a general approach for future studies. Patient-reported HRQOL reflects the patient perspective, and is an information source for health service quality and treatment effectiveness which could support the decision making during reimbursement, access to medicines and health policy process (McKenna, 2011; Calvert et al., 2014). A number of clinical trials include PRO measures for assessing efficacy as endpoint to generate an added value and to support biochemical endpoints (Doward et al., 2010). Post-marketing studies apply PRO measures for evaluation of effectiveness to inform treatment guidelines (Acquadro et al., 2003). To ensure data quality and evidence reporting, the study design and methodology of PRO trials are of importance. PRO-specific rationale and objectives, PRO endpoint specification, timing of PRO assessment, sample size determination for HRQOL studies, PRO data collection, and statistical analysis are key aspects of study design and methodology (Calvert et al., 2014). We developed our study design according to these aspects. It is hoped that this study will provide important information about physical, mental and psycho-social health aspects of TB in the South African socio-demographic context. This additional knowledge about TB, which goes beyond well-known physiological and clinical parameter, should inform current treatment guidelines for South Africa. Currently, a number of new TB drugs and diagnostic devices are under development or in a clinical phase. An integrative knowledge about HRQOL should assist with evaluating the added value of newly developed TB drug products and treatment procedures in comparative effectiveness research (CER) studies. Outcomes from CER will support resource allocation, reimbursement decisions, access to medicines and health policy making in TB in the context of the implementation of National Health Insurance for South Africa. The evaluation of HRQOL and its association with adherence in TB using the selected PRO measures could support future research and development of a TB-specific measure by reviewing the different health domains and their items for TB-relevant aspects. Such TB-specific measure may be integrated into standard care to monitor TB treatment with an integrative patient-centered approach.

### Declarations

#### Ethics Approval and Consent to Participate

The study respects the International Conference on Harmonisation of Technical Requirements for Registration of Pharmaceuticals for Human Use (ICH) guidelines, the Declaration of Helsinki in its current version and South African Good Clinical Practice (GCP). The study was approved by the institutional review commission of the Swiss Tropical and Public Health Institute. Ethical approval and clearance have been obtained from four different institutions: the ethical commission of North-West and Central Switzerland (EKNZ), the ethical committees of the University of Cape Town, City Health City of Cape Town, and the Western Cape Government.

## Author Contributions

TK-H designed the study with support of MS and ES. TK-H, MS, and ES wrote the manuscript. All authors read, contributed to and approved the final manuscript. All authors agree to be accountable for all aspects of the work in ensuring that questions related to the accuracy or integrity of any part of the work are appropriately investigated and resolved.

## Conflict of Interest Statement

The authors declare that the research was conducted in the absence of any commercial or financial relationships that could be construed as a potential conflict of interest.
