# Supplementary material for: How to Evaluate Health-Related Quality of Life and Its Association with Medication Adherence in Pulmonary Tuberculosis – Designing a Prospective Observational Study in South Africa
Source: Front Pharmacol. 2016 May 31;7:125. doi: 10.3389/fphar.2016.00125 (PMC4886690; doi:10.3389/fphar.2016.00125)
Supplement: Supplementary file 1 [file Data_Sheet_1.PDF]

Appendix

Studies applying PRO instruments in TB	Study Type	Country
Adeyeye et al 2014 (Adeyeye et al., 2014)	cross-sectional	Nigeria
Aggarwal et al 2013 (Aggarwal et al., 2013)	longitudinal	India
Atif et al 2014 (Atif et al., 2014b)	cross-sectional	Malaysia
Atif et al 2014 (Atif et al., 2014a)	longitudinal	Malaysia
Atif et al 2013 (Atif et al., 2013)	cross-sectional	Malaysia
Awaisu et al 2012 (Awaisu et al., 2012)	cross-sectional	Malaysia
Babikako et al 2010 (Babikako et al., 2010)	cross-sectional	Uganda
Balgude et al 2012 (Balgude and Sontakke, 2012)	longitudinal	India
Bauer et al 2013 (Bauer et al., 2013)	systematic Review	NA
Chamla 2004 (Chamla, 2004)	longitudinal	China
Chung et al 2012 (Chung et al., 2012)	longitudinal	Taiwan

Corless et al 2009 (Corless et al., 2009)	cross-sectional	South Africa
Dhingra and Rajpal 2005 (Dhingra and Rajpal, 2005)	longitudinal	India
Dhuria et al 2009 (Dhuria et al., 2009)	longitudinal	India
Dion et al 2004 (Dion et al., 2004)	cross-sectional	Canada
Dujaili et al 2015 (Dujaili et al., 2015)	longitudinal	Iraq
Guo et al 2009 (Guo et al., 2009)	systematic review	NA
Guo et al 2008 (Guo N, 2008)	cross-sectional	Canada
Husain et al 2008 (Husain MO, 2008)	cross-sectional	Pakistan
Issa et al 2009 (Bauer et al., 2013)	cross-sectional	Nigeria
Kittikraisak et ak 2012 (Kittikraisak et al., 2012)	cross-sectional	Thailand
Kruijshaar et al 2010 (Kruijshaar et al., 2010)	longitudinal	UK
Louw et al 2012 (Louw et al., 2012)	cross-sectional	South Africa
Maguire et al 2009 (Maguire et al., 2009)	longitudinal	Indonesia

**Mamani et al 2014 (Mamani et al., 2014)**	**longitudinal**	**Western Iran**
**Marra et al 2008 (Marra et al., 2008)**	**longitudinal**	**Canada**
**Masumoto et al 2014 (Masumoto et al., 2014)**	**cross-sectional**	**Philippines**
**McInerney et al 2007 (McInerney et al., 2007)**	**cross-sectional**	**South Africa**
**Muniyandi et al 2007 (Muniyandi et al., 2007)**	**cross-sectional**	**India**
**Naidoo and Mwaba 2010 (Naidoo and Mwaba, 2010)**	**qualitative**	**South Africa**
**Othman et al 2011 (Othman, 2011)**	**cross-sectional**	**Yemen**
**Pasipanodya et al 2007 (Pasipanodya et al., 2007a)**	**cross-sectional**	**USA**
**Pasipanodya et al 2007 B (Pasipanodya et al., 2007b)**	**cross-sectional**	**USA**
**Peltzer et al 2013 (Peltzer et al., 2013)**	**cross-sectional**	**South Africa**
**Peltzer et al 2012 (Peltzer K, 2012)**	**cross-sectional**	**South Africa**
**Rajeswari et al 2005 (Rajeswari et al., 2005)**	**longitudinal**	**India**

**Ralph et al 2013 (Ralph et al., 2013)**	**longitudinal**	**Indonesia**
**Yin et al 2012 (Yin et al., 2012)**	**cross-sectional**	**China**

Table 1 lists all studies included in the development of study design.

PRO measures applied in assessment of HRQOL	Number of single studies identified	Countries
**Short-Form-36 (SF-36)**	18	Uganda, Canada, China, South Africa, UK, India, Western Iran, Malaysia, USA, India
**World Health Organization´s Quality of Life - BREF (WHOQOL-BREF)**	5	India, Taiwan, Nigeria
**EuroQoL (EQ-5D)**	4	Canada, UK, Thailand, Malaysia
**Beck Depression Inventory (BDI)**	4	South Africa, Turkey, Canada
**Kessler-10**	4	South Africa, Ethiopia
**St. George´s Respiratory Questionnaire (SGRQ)**	4	USA, Indonesia
**Standard Gamble (SG)**	3	Canada, UK, Turkey
**Visual Analogue Scale (VAS)**	3	Canada, Thailand
**General Health Questionnaire 12 (GHQ-12)**	2	Turkey, Nigeria
**Mood Adjective Check List Short Form (MACL**	2	Sweden
**Severe Respiratory Insufficiency Questionnaire (SRI)**	2	Spain, Germany
**SF-6D utility score**	2	Canada

**Sickness Impact Profile (SIP)**	2	Sweden
**State-Trait Anxiety Inventory Short Form (STAI-6)**	2	UK, Turkey
**Center for Epidemiologic Studies Depression Scale (CES-D)**	2	UK, USA
**Duke Health Profile (DUKE)**	2	Colombia, Philippines
**DR-12**	2	India, Yemen
**Hospital Anxiety and Depression Scale (HADS)**	1	Pakistan
**Short-Form 12 (SF-12)**	1	South Africa
**Beck Depression Inventory (BDI) Short Form**	1	South Africa
**Brief Disability Questionnaire (BDQ)**	1	Turkey
**Dysfunctional Analysis Questionnaire (DAQ)**	1	India
**Health Utilities Index 2 (HUI 2)**	1	Canada
**Health Utilities Index 3 (HUI 3)**	1	Canada
**Life Satisfaction Index Z**	1	China
**Mental Health Index (MHI-5)**	1	USA
**Modified Version of SF-36**	1	India
**Modified St. Georges Respiratory Questionnaire**	1	Indonesia
**Present State Examination (PSE)**	1	Nigeria
**Rosenberg Self-Esteem Scale (RSE)**	1	South Africa
**Self-Rating Anxiety Scale (SAS)**	1	China
**Sheehan Disability Scale (SDS)**	1	China

**Social Support Rating Scale (SSRS)**	1	China
**Symptoms Check List (SCL-90)**	1	China
**Voice Handicap Index-10 (VHI-10)**	1	Turkey
**Quality of Life Questionnaire (QLQ)**	1	Turkey
**Short-Form 8 (SF-8)**	1	Philippines
**Medical Research Council (MRC) dyspnea scale**	1	Philippines
**Primary Care PTSD screen**	1	South Africa
**World Health Organization´s Quality of Life - HIV (WHOQOL-HIV)**	1	Ethiopia
**The Functional Assessment of Chronic Illness Therapy-Tuberculosis (FACIT-TB)**	1	Iraq
**Illness Perception Questionnaire (IPQ)**	1	Pakistan
**Patient Health Questionnaire (PHQ-9)**	1	Nigeria

Table 2 lists all HRQOL measures identified and extracted from studies included

PRO measures applied in adherence assessment	Number of studies	Country
**Morisky Medication Adherence Scale (MMAS)**	2	South Africa
**Perceived Nonadherence Scale (ACTG)**	1	South Africa

**TB medication adherence scale (TBMAS)**	1	China

Table 3 lists all adherence measures identified and extracted from published studies included.

PRO HRQOL measures	Pathology/Disease
**Baseline and Transition Dyspnea Indexes (BDI-TDI)**	Pulmonary Disease, Chronic Obstructive ,Respiratory Tract Diseases
**Clinical COPD Questionnaire (CCQ)**	Pulmonary Disease, Chronic Obstructive ,Respiratory Tract Diseases
**Chronic Respiratory Disease Questionnaire Self-Administered Standardized (CRQ-SAS)**	Pulmonary Disease, Chronic Obstructive ,Respiratory Tract Diseases
**Inhaled Corticosteroid Questionnaire (ICQ)**	Pulmonary Disease, Chronic Obstructive ,Respiratory Tract Diseases
**St George's Respiratory Questionnaire (SGRQ)**	Pulmonary Disease, Chronic Obstructive ,Respiratory Tract Diseases
**Asthma Control Diary (ACD)**	Respiratory Tract Diseases
**Asthma Control Questionnaire (ACQ)**	Respiratory Tract Diseases
**Asthma Control Test (ACT)**	Respiratory Tract Diseases
**Asthma Quality of Life Questionnaire (AQLQ)**	Respiratory Tract Diseases
**Community-Acquired Pneumonia Symptom questionnaire (CAP-Sym)**	Respiratory Tract Diseases
**EORTC Quality of Life Questionnaire - Lung Cancer Module (EORTC-QLQ LC13)**	Respiratory Tract Diseases
**Geriatric Depression Scale (GDS)**	Psychiatry/Psychology
**Hospital Anxiety and Depression scale (HADS)**	Psychiatry/Psychology
**Inventory of Depressive Symptomatology (IDS-SR and IDS-C)**	Psychiatry/Psychology
**Montgomery-Asberg Depression Rating Scale**	Psychiatry/Psychology

**(MADRS)**	
**Quick Inventory of Depressive Symptomatology (QIDS-SR and QIDS-C)**	Psychiatry/Psychology
**Quality of Life in Depression Scale (QLDS)**	Psychiatry/Psychology

Table 4 presents 17 instruments for HRQOL assessment in the indications pulmonary, respiratory and psychological diseases and linguistically validated for English in South Africa, accessed from PROQOLID database.

Description of Selected HRQOL and Adherence PROMs

The following section provides a description of the PROMs applied to evaluate HRQOL and medication adherence in TB. Information was derived from PROQOLID database (PROQOLID) for EQ-5D, SF-12v2, SGRQ and HADS and from the developer of MMAS Prof Morisky for the MMAS instrument.

EQ-5D-5L (provided through EuroQol)

EQ-5D (Euroqol) assesses health outcomes and is widely used as a utility index for estimating QALYs in cost-effectiveness studies. EQ-5D comprises 5 domains/items

- Mobility
- Self-care
- Usual activities
- Pain/discomfort
- Anxiety/depression

and one vertical visual analogue scale (VAS 20 cm). Each item allows five levels of severity of response ranging from 'no problems' to´ extreme problems'. Higher scores indicate better HRQOL. The VAS records the respondent's self-rated health on a 20 cm vertical, visual analogue scale with endpoints labelled 'the best health you can imagine' and 'the worst health you can imagine'. This information is used as quantitative measure of health as judged by the individual respondents. Index-based values (utilities) are calculated from EQ-5D-5L by deriving values from country-specific value sets. Such value-sets for EQ-5D-5L are currently under development. A crosswalk between the 5L and 3L of EQ-5D is available for 10 countries, including Zimbabwe as only African country. Value sets for South Africa are not available for any version of EQ-5D. The completion time for EQ-5D takes a few minutes. Reliability, validity and ability to detect change are approved (PROQOLID) A minimal important difference (MID) for EQ-5D-3L is approved with Mean MID = 0.074 (range -0.011 - 0.140) and MID = 7 for VAS scores (Walters, 2005).

Short-Form 12 (SF-12) (Author Ware John E, provided through Quality Metrics)

SF-12 is an abbreviated version of SF-36 containing 12 items over 8 domains:

- Physical functioning (PF) with 2 items
- Role physical (RP) with 2 items
- Bodily pain (BP) with 1 item

- General health (GH) with 1 item
- Vitality (VT) with 1 item
- Social functioning (SF) with 1 item
- Role emotional (RE) with 2 items
- Mental health (MH) with 2 items

Domains can be aggregated into composite summary scores for physical and mental state, referred to as Physical Component Score (PCS-12) and Mental Component Sore (MCS-12). PCS-12 includes PF, RP, BP, and GH domains, MCS-12 include VT, SF, RE and MH domains. Scoring ranges from 0 to 100; greater scores represent better HRQOL. The completion time for SF-12v2 is 2 minutes. Reliability, validity and ability to detect change are approved (PROQOLID). A minimal important difference for SF-12v2 is not defined; however a minimum meaningful difference for SF-36 is mentioned as > 3 point change (Maruish, 2009). SF-36 and SF-12v2 are comparable in their outcomes and a minimum meaningful difference of > 3 points can be considered for SF-12v2 (Maruish ME, 2009).

St. George´s Respiratory Questionnaire (SGRQ) (Author Jones Paul W, provided through The St. George's University of London Medical School)

A disease-specific instrument designed to assess patients with respiratory tract diseases and immune system diseases, especially asthma, pulmonary diseases, and chronic obstructive disease. SGRQ comprises 50 items over 3 domains (symptoms, activity, and impacts on daily life) over two parts. Part I covers symptoms (several scales) and Part II covers activity and impacts on daily life (dichotomous true/false) except the last question (4-point Likert scale). The Symptom Component Score is calculated from questions 1-8, the Activity Component Score is calculated from questions 11 and 15, and the Impacts Component Score is calculated from questions 9-10, 12-14, and 16-17. Scores are scaled from 0 to 100, with higher scores indicating worse HRQOL. The completion time for SGRQ is 10 minutes. Reliability, validity and ability to detect change are approved (PROQOLID). A minimal important difference (MID) for SGRQ is defined as improvement of 4 points on the separate domains and the total score (Jones, 2005).

Hospital Anxiety and Depression Scale (HADS) (Authors Snaith RP and Zigmond AS, provided through GL Assessment)

HADS is an instrument applied in psychology/psychiatry to detect states of anxiety and depression. HADS comprises 14 items over 2 dimensions

- Anxiety with 7 items
- Depression with 7 items

Scores for each item range from 0 to 3, with higher scores indicating worse HRQOL (i.e. more anxiety and depression). The anxiety subscale scoring ragnes from 0-21 (8-10 mild anxiety, 11-14 moderate anxiety, 15-21 severe anxiety). The completion time for HADS is 2-5 minutes. Reliability, validity and ability to detect change are approved (PROQOLID). A minimal important difference (MID) for HADS has not been evaluated. A MID was observed for COPD with a value of 1.5 points corresponding to a change from baseline of 20% and informed by both anchor- and distribution-based methods (Puhan et al., 2008).

Morisky Medication Adherence Scale (MMAS-8) (Author Morisky DE)

MMAS-8 is being used in the assessment of self reported medication taking behaviour (Morisky, 2008a;Krousel-Wood, 2009;Morisky and DiMatteo, 2011). The scale is a generic measure assessing long-term chronic and infectious medical regimens, such as high blood pressure, diabetes, tuberculosis, HIV, elevated serum lipids, osteoporosis, immuno-suppressant medication. It is a reliable and valid indicator to assess self reported medication-taking behaviour, including several levels of criterion related validity (blood pressure control, HgA1c) discriminant validity (social desirability) and persistence using pharmacy fills as a criterion. MMAS has a high sensitivity of 93% to identify low adherence and is a simple scale to identify and monitor adherence (Morisky, 2008b). The MMAS-8 scale ranges from 0 to 8.0; total scores are interpreted in the following way:

- Low adherence <6.0
- Medium adherence 6.0 – 8.0
- High adherence =8.0

Use of the ©MMAS is protected by US copyright laws. Permission for use is required. A license agreement is available from: Donald E. Morisky, ScD, ScM, MSPH, Professor, Department of Community Health Sciences, UCLA School of Public Health, 650 Charles E. Young Drive South, Los Angeles, CA 90095-1772.

Reference List

Adeyeye, O.O., Ogunleye, O.O., Coker, A., Kuyinu, Y., Bamisile, R.T., Ekrikpo, U., and Onadeko, B. (2014). Factors influencing quality of life and predictors of low quality of life scores in patients on treatment for pulmonary tuberculosis: a cross sectional study. *Journal of Public Health in Africa* 5. doi: 10.4081/jphia.2014.366.

Aggarwal, A.N., Gupta, D., Janmeja, A.K., and Jindal, S.K. (2013). Assessment of health-related quality of life in patients with pulmonary tuberculosis under programme conditions. *Int J Tuberc Lung Dis* 17**,** 947-953. doi: 10.5588/ijtld.12.0299.

Atif, M., Sulaiman, S.A., Shafie, A.A., Asif, M., and Ahmad, N. (2013). SF-36v2 norms and its' discriminative properties among healthy households of tuberculosis patients in Malaysia. *Qual Life Res* 22**,** 1955-1964. doi: 10.1007/s11136-012-0337-x.

Atif, M., Sulaiman, S.A., Shafie, A.A., Asif, M., Sarfraz, M.K., Low, H.C., and Babar, Z.U. (2014a). Impact of tuberculosis treatment on health-related quality of life of pulmonary tuberculosis patients: a follow-up study. *Health Qual Life Outcomes* 12**,** 19. doi: 10.1186/1477-7525-12-19.

Atif, M., Toghrayee, Z., Sulaiman, S.A.S., Shafie, A.A., Low, H.C., and Babar, Z.-U.-D. (2014b). Missing Data Analysis in Longitudinal Studies: Findings from a Quality of

Life Study in Malaysian Tuberculosis Patients. *Applied Research in Quality of Life* 10**,** 95-105. doi: 10.1007/s11482-014-9302-x.

Awaisu, A., Haniki Nik Mohamed, M., Noordin, N.M., Muttalif, A.R., Aziz, N.A., Syed Sulaiman, S.A., and Mahayiddin, A.A. (2012). Impact of connecting tuberculosis directly observed therapy short-course with smoking cessation on health-related quality of life. *Tob Induc Dis* 10**,** 2. doi: 10.1186/1617-9625-10-2.

Babikako, H.M., Neuhauser, D., Katamba, A., and Mupere, E. (2010). Feasibility, reliability and validity of health-related quality of life questionnaire among adult pulmonary tuberculosis patients in urban Uganda: cross-sectional study. *Health Qual Life Outcomes* 8**,** 93. doi: 10.1186/1477-7525-8-93.

Balgude, A., and Sontakke, S. (2012). Study of impact of antitubercular therapy on quality of life. *Indian J Med Sci* 66**,** 71-77. doi: 10.4103/0019-5359.110911.

Bauer, M., Leavens, A., and Schwartzman, K. (2013). A systematic review and meta-analysis of the impact of tuberculosis on health-related quality of life. *Qual Life Res* 22**,** 2213-2235. doi: 10.1007/s11136-012-0329-x.

Chamla, D. (2004). The assessment of patients' health-related quality of life during tuberculosis treatment in Wuhan, China. *Int J Tuberc Lung Dis* 8**,** 1100-1106.

Chung, W.S., Lan, Y.L., and Yang, M.C. (2012). Psychometric testing of the short version of the world health organization quality of life (WHOQOL-BREF) questionnaire among pulmonary tuberculosis patients in Taiwan. *BMC Public Health* 12**,** 630. doi: 10.1186/1471-2458-12-630.

Corless, I.B., Wantland, D., Bhengu, B., McInerney, P., Ncama, B., Nicholas, P.K., McGibbon, C., Wong, E., and Davis, S.M. (2009). HIV and tuberculosis in Durban, South Africa: adherence to two medication regimens. *AIDS Care* 21**,** 1106-1113. doi: 10.1080/09540120902729932.

Dhingra, V.K., and Rajpal, S. (2005). Health related quality of life (HRQL) scoring (DR-12 score) in tuberculosis--additional evaluative tool under DOTS. *J Commun Dis* 37**,** 261-268.

Dhuria, M., Sharma, N., Narender Pal, S., Ram Chander, J., Saha, R., and Gopal Krishan, I. (2009). A study of the impact of tuberculosis on the quality of life and the effect after treatment with DOTS. *Asia Pac J Public Health* 21**,** 312-320. doi: 10.1177/1010539509336242.

Dion, M.J., Tousignant, P., Bourbeau, J., Menzies, D., and Schwartzman, K. (2004). Feasibility and reliability of health-related quality of life measurements among tuberculosis patients. *Qual Life Res* 13**,** 653-665.

Dujaili, J.A., Sulaiman, S.A., Hassali, M.A., Awaisu, A., Blebil, A.Q., and Bredle, J.M. (2015). Health-related quality of life as a predictor of tuberculosis treatment outcomes in Iraq. *Int J Infect Dis* 31**,** 4-8. doi: 10.1016/j.ijid.2014.12.004.

Euroqol *EQ-5D-5L* [Online]. Available: http://www.euroqol.org/eq-5d-products/eq-5d-5l.html [Accessed 16 December 2015].

Guo, N., Marra, F., and Marra, C.A. (2009). Measuring health-related quality of life in tuberculosis: a systematic review. *Health Qual Life Outcomes* 7**,** 14. doi: 10.1186/1477-7525-7-14.

Guo N, Marra, C., Marra F, Moadebi S, Elwood RK, FitzGerald JM (2008). Health state utilities in latent and active tuberculosis. *Value in Health* 11**,** 1154-1161.

Husain MO, Dearman, S., Chaudhry IB, Rizvi N, Waheed W (2008). The relationship between anxiety, depression and illness perception in tuberculosis patients in Pakistan. *Clinical Practice and Epidemiology in Mental Health* 4**,** 1-5. doi: 10.1186/1745-0179-44.

Jones, P.W. (2005). St. George´s Respiratory Questionnaire: MCID. *Journal of Chronic Obstructive Pulmonary* 2**,** 75-79. doi: 10.1081/COPD-200050513.

Kittikraisak, W., Kingkaew, P., Teerawattananon, Y., Yothasamut, J., Natesuwan, S., Manosuthi, W., Chongsuvivatwong, V., and Whitehead, S.J. (2012). Health related quality of life among patients with tuberculosis and HIV in Thailand. *PLoS One* 7**,** e29775. doi: 10.1371/journal.pone.0029775.

Krousel-Wood, M., Islam, T., Webber, L.S., Re, R.N., Morisky, D.E., Muntner, P. (2009). New Medication Adherence Scale Versus Pharmacy Fill Rates in Seniors with Hypertension. *THE AMERICAN JOURNAL OF MANAGED CARE* 15.

Kruijshaar, M.E., Lipman, M., Essink-Bot, M.L., Lozewicz, S., Creer, D., Dart, S., Maguire, H., and Abubakar, I. (2010). Health status of UK patients with active tuberculosis. *Int J Tuberc Lung Dis* 14**,** 296-302.

Louw, J., Peltzer, K., Naidoo, P., Matseke, G., McHunu, G., and Tutshana, B. (2012). Quality of life among tuberculosis (TB), TB retreatment and/or TB-HIV co-infected primary public health care patients in three districts in South Africa. *Health Qual Life Outcomes* 10**,** 77. doi: 10.1186/1477-7525-10-77.

Maguire, G.P., Anstey, N.M., Ardian, M., Waramori, G., Tjitra, E., Kenangalem, E., Handojo, T., and Kelly, P.M. (2009). Pulmonary tuberculosis, impaired lung function, disability and quality of life in a high-burden setting. *Int J Tuberc Lung Dis* 13**,** 1500-1506.

Mamani, M., Majzoobi, M.M., Ghahfarokhi, S.M., Esna-Ashari, F., and Keramat, F. (2014). Assessment of Health-related Quality of Life among Patients with Tuberculosis in Hamadan, Western Iran. *Oman Med J* 29**,** 102-105. doi: 10.5001/omj.2014.25.

Marra, C.A., Marra, F., Colley, L., Moadebi, S., Elwood, R.K., and Fitzgerald, J.M. (2008). Health-related quality of life trajectories among adults with tuberculosis: differences between latent and active infection. *Chest* 133**,** 396-403. doi: 10.1378/chest.07-1494.

Maruish, M.E., and Kosinski, M. (2009). "A Guide to the Development of Certified Short Form Survey Interpretation and Reporting Capabilities". Quality Metrics Incorporated).

Maruish, M.E., and Kosinski, M. (2009). "A guide to the development of certified short form survey interpretation and reporting capabilities ", (ed.) Q. Incorporated.).

Masumoto, S., Yamamoto, T., Ohkado, A., Yoshimatsu, S., Querri, A.G., and Kamiya, Y. (2014). Factors associated with health-related quality of life among pulmonary tuberculosis patients in Manila, the Philippines. *Qual Life Res* 23**,** 1523-1533. doi: 10.1007/s11136-013-0571-x.

McInerney, P.A., Nicholas, P.K., Wantland, D., Corless, I.B., Ncama, B., Bhengu, B., McGibbon, C.A., Davis, S.M., and Gallagher, D.M. (2007). Characteristics of anti-tuberculosis medication adherence in South Africa. *Appl Nurs Res* 20**,** 164-170. doi: 10.1016/j.apnr.2006.06.006.

Morisky, D.E., Ang, A., Krousel-Wood, M., Ward, H.J. (2008a). Predictive Validation of a Medication Adherence Measure in an Outpatient Setting. *The Journal of Clinical Hypertension* 10**,** 348-354.

Morisky, D.E., Ang, A., Krousel-Wood, M., Ward, H.J. (2008b). Predictive Validity of a Medication Adherence Measure in an Outpatient Setting. *The Journal of Clinical Hypertension* 10**,** 348-354.

Morisky, D.E., and DiMatteo, M.R. (2011). Improving the measurement of self-reported medication nonadherence: Final response. *Journal of Clinical Epidemiology* 64**,** 262-263. doi: 10.1016/j.jclinepi.2010.09.010.

Muniyandi, M., Rajeswari, R., Balasubramanian, R., Nirupa, C., Gopi, P.G., Jaggarajamma, K., Sheela, F., and Narayanan, P.R. (2007). Evaluation of post-treatment health-related quality of life (HRQoL) among tuberculosis patients. *Int J Tuberc Lung Dis* 11**,** 887-892.

Naidoo, P., and Mwaba, K. (2010). Helplessness, Depression, and Social Support Among People Being Treated for Tuberculosis in South Africa. *Social Behavior and Personality: an international journal* 38**,** 1323-1333. doi: 10.2224/sbp.2010.38.10.1323.

Othman, Q. (2011). Health related quality of life of pulmonary and extrapulmonary tuberculosis patients in Yemen. *African Journal of Pharmacy and Pharmacology* 5**,** 547-553. doi: 10.5897/ajpp11.078.

Pasipanodya, J.G., Miller, T.L., Vecino, M., Munguia, G., Bae, S., Drewyer, G., and Weis, S.E. (2007a). Using the St. George respiratory questionnaire to ascertain health quality in persons with treated pulmonary tuberculosis. *Chest* 132**,** 1591-1598. doi: 10.1378/chest.07-0755.

Pasipanodya, J.G., Miller, T.L., Vecino, M., Munguia, G., Garmon, R., Bae, S., Drewyer, G., and Weis, S.E. (2007b). Pulmonary impairment after tuberculosis. *Chest* 131**,** 1817-1824. doi: 10.1378/chest.06-2949.

Patient-reported Outcomes and Quality of Life Database (PROQOLID). Mapi Research Institute. [Online] Available: www.proqolid.org. [Accessed: 12 Feb 2014].

Peltzer, K., Naidoo, P., Matseke, G., Louw, J., McHunu, G., and Tutshana, B. (2013). Prevalence of post-traumatic stress symptoms and associated factors in tuberculosis (TB), TB retreatment and/or TB–HIV co-infected primary public health-care patients in three districts in South Africa. *Psychology, Health & Medicine* 18**,** 387-397. doi: 10.1080/13548506.2012.726364.

Peltzer K, N.P., Matseke G, Louw J, McHunu G, Tutshana B (2012). Prevalence of psychological distress and associated factors in tuberculosis patients in public primary care clinics in South Africa. *BMC Psychiatry* 12**,** 1-10.

Puhan, M.A., Frey, M., Buchi, S., and Schunemann, H.J. (2008). The minimal important difference of the hospital anxiety and depression scale in patients with chronic obstructive pulmonary disease. *Health Qual Life Outcomes* 6**,** 46. doi: 10.1186/1477-7525-6-46.

Rajeswari, R., Muniyandi, M., Balasubramanian, R., and Narayanan, P.R. (2005). Perceptions of tuberculosis patients about their physical, mental and social well-being: a field report from south India. *Soc Sci Med* 60**,** 1845-1853. doi: 10.1016/j.socscimed.2004.08.024.

Ralph, A.P., Kenangalem, E., Waramori, G., Pontororing, G.J., Sandjaja, Tjitra, E., Maguire, G.P., Kelly, P.M., and Anstey, N.M. (2013). High morbidity during treatment and residual pulmonary disability in pulmonary tuberculosis: under-recognised phenomena. *PLoS One* 8**,** e80302. doi: 10.1371/journal.pone.0080302.

Walters, S.J., Brazier, J.E. (2005). Comparison of the minimally important difference for two health state utility measures: EQ-5D and SF-6D. *Quality of Life Research* 14**,** 1523-1532. doi: 10.1007/s11136-004-7713-0.

Yin, X., Tu, X., Tong, Y., Yang, R., Wang, Y., Cao, S., Fan, H., Wang, F., Gong, Y., Yin, P., and Lu, Z. (2012). Development and validation of a tuberculosis medication adherence scale. *PLoS One* 7**,** e50328. doi: 10.1371/journal.pone.0050328.
