# Supplementary material for: How to Evaluate Health-Related Quality of Life and Its Association with Medication Adherence in Pulmonary Tuberculosis – Designing a Prospective Observational Study in South Africa
Source: Front Pharmacol. 2016 May 31;7:125. doi: 10.3389/fphar.2016.00125 (PMC4886690; doi:10.3389/fphar.2016.00125)
Supplement: Supplementary file 2 [file Data_Sheet_2.PDF]

Standard Operating Procedure (SOP) for application of questionnaires evaluating the health-related quality of life and medication adherence in pulmonary tuberculosis

1. Purpose and Scope

- The present SOP provides an overview of the procedures that take place during application of questionnaires evaluating the health-related quality of life (HRQOL) and medication adherence in patients newly diagnosed with pulmonary tuberculosis. The SOP refers to the research study entitled *health-related quality of life and its association to medication adherence in pulmonary tuberculosis in South Africa – an integrated patient-centred outcomes approach* (protocol version 1.4/25.08.2014).
- The applied questionnaires are objective means of collecting information about TB patient´s HRQOL and medication adherence behavior. In the present research study they are used as independent research instruments.
- There are no strict reporting criteria for questionnaire-based research projects, when compared with the conduct of randomized trials. In order, therefore, to prevent methodological errors and to promote the collection of high quality data a process should be followed to ensure that questionnaire-based research and data collection is well-designed, well-managed, non-discriminatory and reduces potential bias, with a view to contributing to a generalizable evidence base.
- This SOP is relevant for all research team members involved in the data collection process and application of questionnaires evaluating HRQOL and medication adherence in pulmonary TB patients. It is important that all study personnel fully understand and comply with this SOP.

2. Definitions

This section provides a short explanation of terminology used in this SOP.

**Terminology**	**Explanation**
Face-to-face administration	In-person administration of a questionnaire by a trained interviewer
Health-related quality of life	The value assigned to duration of life as modified by the impairments, functional states, perceptions, and social opportunities that are influenced by disease, injury, treatment, or policy. Measurement of health-related quality of life addresses the various dimensions of health and well-being
Measure	Questionnaires designed for use with a particular patient group
Medication adherence	Patient behavior with regard to the prescribed interval, dose, and dosing regimen as well as quality of how medication is taken and is expressed as percentage of total number of doses taken or therapy days available

Patient-reported outcome	Any report of the status of a patient's health condition that comes directly from the patient, without interpretation of the patient's response by a clinician or anyone else
Self-administered	Respondents read and answer the questions by themselves, without assistance. The respondent completes the questionnaire herself or himself according to written or oral instructions

3. Abbreviations

HRQOL	Health-related quality of life
PRO	patient-reported outcome
TB	Tuberculosis

4. Roles and Responsibilities

- This SOP applies to research team members of the present research study, who are involved in the data collection process including the application of questionnaires evaluating HRQOL and medication adherence
- Research team members directly involved in the data collection process comprise:
  - Main researcher
  - Principal investigators (research supervisors)
  - Research assistant
  - Assisting field worker

5. Data Protection and Confidentiality

- The collection and processing of personal or sensitive data is governed by local law of South Africa
- In the majority of cases where sensitive personal data are being collected, explicit consent is necessary. For consent to be explicit, individuals must have a full understanding of what their data will be used for and they must 'opt-in.' A patient information and informed consent document is handed out to the patient and the informed consent has to be signed after agreement to participate. The patient information remains with the patient; the signed informed consent is collected and remains with the research team
- Personally sensitive data include, but are not limited to, socio-demographic data, physical and mental health, social behaviour, and clinical data in the context to anti-TB drug treatment monitoring
- Personally sensitive data are protected by anonymization of each patient participating. A unique identifier code is assigned by the investigator to each study participant. This subject identification (SID) code protects the subject's identity and confidentiality in the research file. SID is used in lieu of the subject's name on all

research documents that go to the sponsor or outside of the institution where the patient is treated

- The subject identification (SID) code is additionally collected on a subject identification code list. The SID code list exists for each study site facility and comprises the SID code for each patient enrolled into the study at the specific study site and links the patient SID code to the medical record number of the patient at the clinic. Since one research team member is dedicated to one health facility, the SID code list per clinic remains with the respective research team member during the time of recruitment. After recruitment the SID code list is handed over to the main researcher. During recruitment the field worker has to show the SID code list to the main researcher or to the research assistant on a weekly basis.

6. Questionnaires and Study Material

The questionnaires applied in the present research study comprise

- Four different HRQOL measures:
  - Short Form 12 (SF-12)
  - EuroQol 5 Dimensions (EQ-5D-5L)
  - St. George´s Respiratory Questionnaire (SGRQ)
  - Hospital Anxiety and Depression Scale (HADS)
- One adherence medication measure:
  - Morisky Medication Adherence Scale 8 items (MMAS-8)
- One questionnaire for assessing socio-demographic characteristics

All questionnaires are PRO measures which are paper-based and self-administered, thereby providing subjective information from the patient directly. All questionnaires are closed which means each item is answered by ticking the most applicable predetermined answer.

Further study material comprises

- Patient Information and Informed Consent
- Subject Identification Code List
- Monitoring Sheet

The patient information is a document that explains the study research in a more common language. This document is designed for the patient to take it home for his or her reference. The informed consent is a one-pager available in English and Xhosa and has to be signed by the patient if he or she confirms to participate in the study. The informed consent has to be collected and handed over to the main researcher or principal investigator. The monitoring sheet is a one-pager assigned for each participating patient. This sheet is a constant part of the patient research folder and is used for every visit you interact with the patient. The monitoring sheet contains information about facility name and study site log number, patient

code (SID code), patient study group, treatment start, expected follow-up visits (based on treatment start), and sputum smear results at all visits where questionnaires are applied.

7. Procedures

7.1 Eligible participants

- The purpose of application of questionnaires is to evaluate the HRQOL and medication adherence of pulmonary TB patients during anti-TB treatment comprising four different antibiotic drugs over 6 month.
- Patients eligible for the research study are older than 18 years, are newly diagnosed with pulmonary TB and will start standard drug treatment for their first time; and who have no HIV co-infection and no multi-drug resistant (MDR) TB.
- Eligible patients belong either to a group of patients receiving complete treatment under supervision in the health clinic – thereafter called" clinic group"; or eligible patients belong to a group of patients receiving their treatment in their community and visiting the health clinic on predefined visits for diagnostic monitoring and drug collection – thereafter called "community group"
- All eligible patients participating (clinic group and community group) fill out four different HRQOL questionnaires and one socio-demographic questionnaire at baseline (visit 0)
- All eligible patients participating (clinic group and community group) fill out four different HRQOL questionnaires and one medication adherence questionnaire at visit 1 (after 4 weeks of treatment), visit 2 (after 8 weeks of treatment and changing from intensive to continuous phase), visit 3 (after 16 weeks of treatment), and visit 4 (after 24 weeks treatment and end of treatment).

7.2 Data collection process

- Data collection comprises a time frame of 6 month of anti-TB drug treatment comprising four different antibiotics for each eligible participant, starting with treatment start, thereafter called baseline, and finalizing with end of treatment
- Data are collected at five different time points (baseline, visits 1,2,3,4) for eligible participants from the clinic group and at a minimum of three different time points (baseline, visit 2, visit 4) from eligible participants from the community group
- The five data collection points are defined as in the following table:

Data Collection Time Point	Description
Visit 0	begin of treatment which is start of the intensive phase and baseline (BL)
Visit 1	after 1 month of treatment and middle of the intensive phase
Visit 2	after 2 month of treatment and switch from intensive to continuation phase
Visit 3	after 4 month of treatment which is in the middle of the continuation phase
Visit 4	after 6 month of treatment which is end of treatment (EOT)

- At baseline socio-demographic characteristics are collected

- At baseline and at visits 1, 2, 3, and 4 HRQOL data are collected
- At visit 2 (change from intensive to continuous phase) and visit 4 (end of treatment) medication adherence data are collected
- The administration of all questionnaires for data collection is assigned to pre-determined research team members

Data collection at baseline has a time window of three days, meaning data can be collected within first three days of treatment start. All follow-up visits have a time window of one week.

7.3 Data Collection and Questionnaire Administration

Step-wise process at Baseline:

Step 1: A nurse informs you via SMS or by calling that the clinic has identified an eligible patient; the nurse provides you with the date the respective patient is visiting the clinic. You have to be at the clinic at the same date.

Step 2: A nurse refers you to the eligible patient. Before you talk to the patient, ask the nurse to hand out a safety mask for you. You have to wear a safety mask all the time you talk to patients for your own health safety.

Step 3: Introduce the study to the eligible patient by using the patient information document; mention that participants are receiving a token in form of a voucher from us after completion of each visit. After he or she has confirmed to participate in the study, hand out the patient information to the patient for their reference.

Step 4: Get the informed consent form signed by the patient and collect the informed consent for our reference.

Step 5: Use the SID code list (a separate document) and write down a patient unique SID code; the SID coding for patients is linked to the study site log number. Study site log numbers are the following:

Study Site Log Number	Study Site Name
1-HRQOLTB	Khayelitsha Site B
2-HRQOLTB	Michael Mapongwana Hospital
3-HRQOLTB	Town 2 Clinic
4-HRQOLTB	Kuyasa Clinic
5-HRQOLTB	Matthew Goniwe Clinic
6-HRQOLTB	Nolungile Clinic

The unique patient SID code is composed of the study site log number plus a consecutive numbering; the following is an example of a consecutive numbering for Khayelitsha site B:

1-HRQOLTB_001
1-HRQOLTB_002
1-HRQOLTB_003

Step 6: Use the SID code list to write down the medical record number of the patient which the study site has assigned to the patient in the TB registry book.

Step 7: Use the monitoring sheet (a separate document) to write down

- Facility name and study site log number
- Patient code (SID code)
- Patient study group (clinic group or community group)
- Date of treatment start
- Confirm if participant has received a voucher after questionnaire completion or not with YES or NO
- Sputum smear result at baseline/treatment start

Ask the nurse to provide you with the date of treatment start which is written down in the TB registry book and with the sputum smear result before treatment.

Step 8: Start with the baseline interviews by applying the document “Questionnaires at baseline”. Apply the questionnaires to the patient while he or she is waiting for seeing the doctor and /or waiting for treatment/diagnostic results.
The questionnaires are originally designed for self-administration and the patient should complete the questionnaires without the help of others (e.g. site staff, family, friends, etc.). If the patient is not illiterate the patient should has the choice of completing the questionnaires by their own.
The *only* exception: if the patient is blind or illiterate the questionnaires may be read to the patient verbatim, but the reader must not aid in the interpretation of questions or in the selection of answers.
All questionnaires should be completed before any other site activities, i.e. before seeing the physician, any tests or treatment, receive results of tests, etc.

Step 9: Explain shortly the significance and relevance of the questionnaires to the participant; remind participants that we are asking them to complete these questionnaires because we are interested in hearing directly from them how they are doing; this will help motivate participants to comply with data collection.

Step 10: If the patient self-completes the questionnaires make sure the patient understands how to fill out the questionnaires and make sure they can be completed in privacy. Inform the patient that the information is confidential.

Step 11: The person responsible for questionnaires (assigned research team members) should retrieve the completed forms. Check that all questions have been answered and that only one answer is recorded for each question.

If a participant has questions or is unable to complete the questionnaires by himself/ herself:

- Remind him/her there are no right or wrong answers
- Remind him/her to choose the answer that most reflects what is true for them
- Do NOT reword, interpret or paraphrase questions or response options
- Do NOT suggest answers or help a patient select an answer under any circumstances

Step-wise process for follow-up visits:

Step 1: Use the monitoring sheet (a separate document) to write down:

- Visit date according to clinic or community group
- Confirm if participant has received a voucher after questionnaire completion or not with YES or NO
- Sputum smear result according to the visit date

Step 2: Apply the questionnaires to the patient while he or she is waiting for seeing the doctor and /or waiting for treatment/diagnostic results.
The questionnaires are originally designed for self-administration and the patient should complete the questionnaires without the help of others (e.g. site staff, family, friends, etc.). If the patient is not illiterate the patient should has the choice of completing the questionnaires by their own.
The *only* exception: if the patient is blind or illiterate the questionnaires may be read to the patient verbatim, but the reader must not aid in the interpretation of questions or in the selection of answers.
All questionnaires should be completed before any other site activities, i.e. before seeing the physician, any tests or treatment, receive results of tests, etc.

Step 3: Explain shortly the significance and relevance of the questionnaires to the participant; remind participants that we are asking them to complete these questionnaires because we are interested in hearing directly from them how

they are doing; this will help motivate participants to comply with data collection.

Step 4: If the patient self-completes the questionnaires make sure the patient understands how to fill out the questionnaires and make sure they can be completed in privacy. Inform the patient that the information is confidential.

Step 5: The person responsible for questionnaires (assigned research team members) should retrieve the completed forms. Check that all questions have been answered and that only one answer is recorded for each question.

If a participant has questions or is unable to complete the questionnaires by himself/ herself:

- Remind him/her there are no right or wrong answers
- Remind him/her to choose the answer that most reflects what is true for them
- Do NOT reword, interpret or paraphrase questions or response options
- Do NOT suggest answers or help a patient select an answer under any circumstances

7.4 Missing Data

- Quickly review the completed questionnaires to look for missing or multiple responses to a question
- Patients should be approached as quickly as possible if any responses are missing or are marked incorrectly and asked to complete all questions and/or clarify responses where more than one response has been given
- PRO data cannot be queried at a later date
- Missing questionnaires and missing questions should be minimized as much as possible
- If there is much missing data we will not be able to meaningfully interpret the PRO data

8. Related Documents

- PhD proposal version 1.4/25.08.2014
- Questionnaires version 1.2/15.08.2014
- Patient Information and Informed Consent version 1.2/11.08.2014
- Monitoring Sheet version 1.0/15.10.2014
- Subject Identification Code List version 1.0/15.10.2014
